# PDIM/PGL-deficient *Mycobacterium marinum* shows disrupted protein secretion measured with bottom-up label-free quantitative analysis in data-dependent acquisition mode on a timsTOF Pro 2 mass spectrometer

**DOI:** 10.1128/mra.01161-25

**Published:** 2025-12-29

**Authors:** Simon D. Weaver, Daniel D. Hu, Bradley S. Jones, Patricia A. Champion, Matthew M. Champion

**Affiliations:** 1 Department of Chemistry and Biochemistry, University of Notre Dame 6111, Notre Dame, Indiana, USA; 2 Department of Biological Sciences, University of Notre Dame 6111, Notre Dame, Indiana, USA; 3 Eck Institute for Global Health, University of Notre Dame, Notre Dame, Indiana, USA; University of Pittsburgh School of Medicine, Pittsburgh, Pennsylvania, USA

**Keywords:** mycobacteria, PDIM/PGL, proteomics, Secretome, ESX-1

## Abstract

We investigated the chemical and functional consequences of a *Mycobacterium marinum* strain deficient in phthiocerol dimycocerosate and phenolic glycolipid (PDIM/PGL) virulence lipids using quantitative proteomics. The significant dysregulation of 1,006 proteins and altered ESAT-6 System-1 export and virulence were measured. PDIM/PGL deficiency specifically decreased the export of proteins with low isoelectric points.

## ANNOUNCEMENT

Human tuberculosis is a disease of global concern caused by *Mycobacterium tuberculosis* (1). *Mycobacterium marinum* is an established model to study *M. tuberculosi*s virulence (2–9). The conserved ESAT-6 system-1 (ESX-1) secretion system is essential for virulence (10, 11). ESX-1 and the virulence lipids phthiocerol dimycocerosate and phenolic glycolipid (PDIM/PGL) are important for phagosome lysis, but the impact of these lipids on protein secretion was unknown (12–14). The Mas protein is required for the biosynthesis of PDIM/PGL lipids (15–17). We investigated a Δ*mas M. marinum* strain (along with its complement Δ*mas*/p*mas*) to define the impact of PDIM/PGL on protein secretion. The Δ*mas* strain is attenuated and deficient in PDIM/PGL biosynthesis (16, 18–20). Using standard quantitative proteomics, we measured significant changes in the secretion of the ESX-1 substrates, EspE and EspF, which are known virulence factors (21). Additionally, Δ*mas* showed a general secretion defect not observed in ESX-1-deficient strains (22). We hypothesized that in addition to specific effects on ESX-1 substrates, loss of PDIM/PGLs causes other secretory deficiencies in mycobacteria. This announcement identifies characteristics of the proteins that are dependent on PDIM/PGL for secretion.

*M. marinum* strains, wild-type (WT, M strain, ATCC BAA-535), Δ*mas*, and Δ*mas*/*pmas* were grown in Middlebrook 7H9 with 0.1% Tween-80. Cultures were diluted to an OD_600_ of 0.8 in Sauton’s media with 0.01% Tween-80. After growth for 48 hours at 30°C, secreted protein fractions were collected for proteomic analysis (23, 24). Tryptic digests were performed on 100 µg of protein using silica filter traps (25) and were desalted by HLB solid-phase extraction. Nine replicates (100 ng) per strain were analyzed on an Evosep One and timsTOF Pro 2 LC-MS/MS system using 15 spd on a 150 µm × 150 mm PepSep-C_18_ column. Instrument settings were identical to those described: 100–1,700 *m*/*z* and a resolution of 50,000 at 1,200 *m*/*z* (FWHM) (23). Raw files were searched using PEAKS Online X search engine (build 1.4.2020-10-21_171258) as described (23). *M. marinum* M strain FASTA was from Mycobrowser (v.4-5453 entries) (26). Protein abundances were imported to R (v.4.3.2); QFeatures (v.1.10.0) and limma (v.3.56.2) (27) were used to perform differential expression analysis. Molecular weight, isoelectric point (pI), and GRAVY (hydrophobicity) were calculated using Peptides (v.2.4.5). Gene Ontology (GO) analysis was performed with DAVID (v.2021) (28) using default terms.

**Fig 1:**
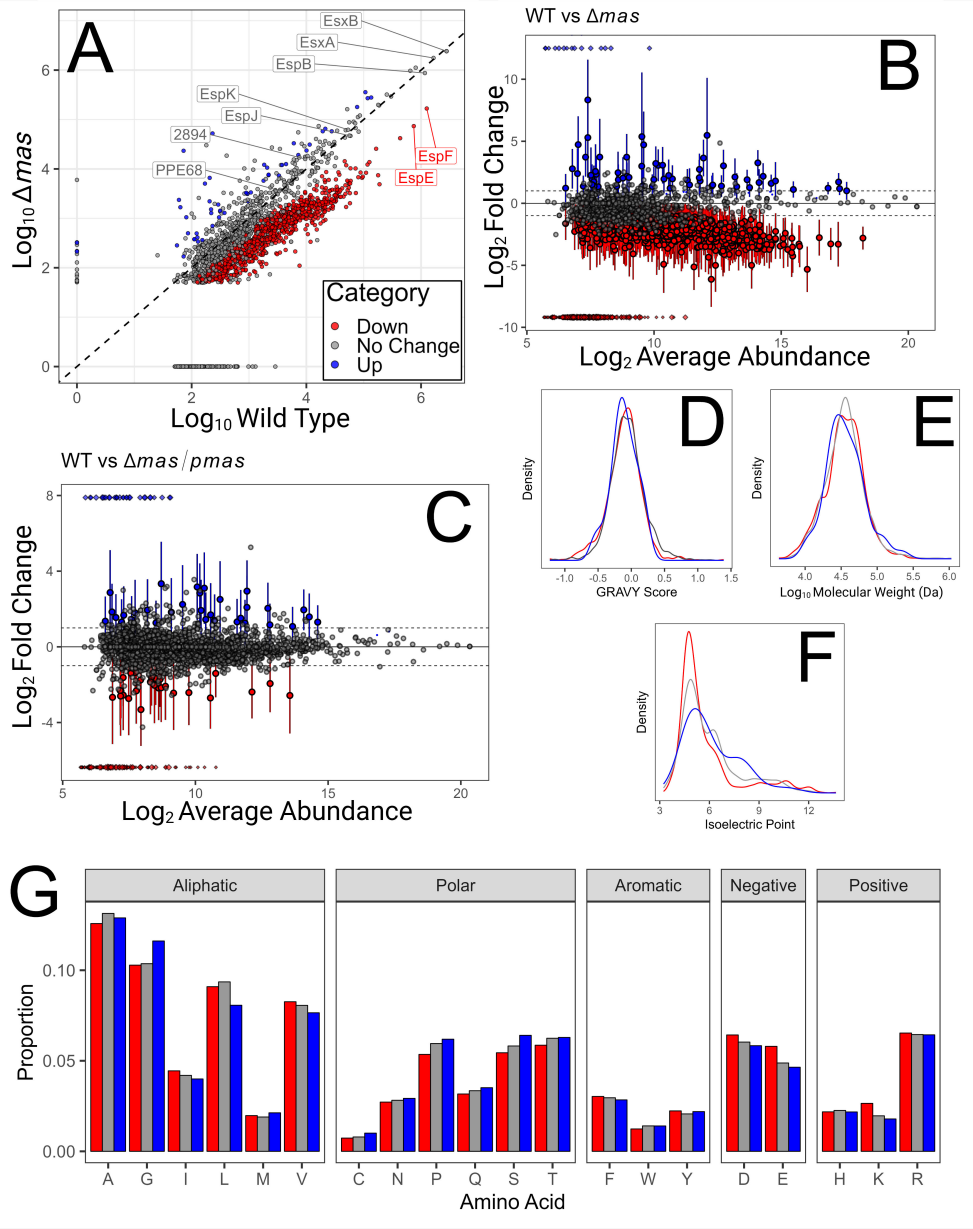
Analysis of the secretome in a PDIM/PGL-deficient strain (Δ*mas*) of *M. marinum*. (**A**) Protein abundance in the secreted proteomes of WT and Δ*mas* plotted as a scatter with WT on the *x*-axis and Δ*mas* on the *y*-axis. ESX-1 substrates are labeled, showing a decrease in secretion of EspE and EspF and no significant change in the other substrates (labeled in gray) (22). Proteins with significant changes in secretion are highlighted in blue (increased) and red (decreased) with filters as described in panels B and C. (**B**) An M-A plot showing proteins with significant (B-H, *P* value <0.05) increased (blue) and decreased (red) secretion from the Δ*mas* strain compared to the wild-type (WT) strain with an absolute log_2_ fold change of at least 1. Infinite values are plotted at the top and bottom of the plot. The *x*-axis shows average protein abundance between the two strains. Error bars show 95% confidence intervals calculated after a B-H correction for significantly changing proteins. (**C**) Same as panel B but comparing Δ*mas*/p*mas* to WT. (**D–F**) Density plots of the protein groups as assigned in panel A showing (**D**) GRAVY score, (**E**) molecular weight, and (**F**) isoelectric point. (**G**) Amino acid composition of the proteins in the groups as assigned in panel A.

We observed a nominally bimodal distribution of secreted protein abundances between the WT and Δ*mas* strains. One distribution appeared unchanged, including most ESX-1 substrates, and one distribution showed decreased secretion, including EspE and EspF (Fig. 1A). We hypothesized that the differential PDIM/PGL-dependent secretion of these two populations of proteins was due to distinct physicochemical properties. Differential expression (*limma*) categorized proteins as up (LFC >1, B-H *P* value <0.05), down (LFC <−1, B-H *P* value <0.05), or unchanged (all other proteins) (Fig. 1B). We repeated the same comparison between WT and Δ*mas*/*pmas* to exclude false positives (60 out of 2,284) (Fig. 1C). After this analysis, the secretion of 973 proteins was significantly reduced, and the secretion of 33 proteins was significantly increased in the Δ*mas* strain from a total of 2,170 detected proteins (Fig. 1A), consistent with the observed bimodal distribution.

GO analysis of the downregulated secretome compared to all detected proteins showed no significant enrichment for any biological processes or molecular functions. The molecular weight and GRAVY score distributions of these groups were essentially identical (Fig. 1D–E). However, those proteins with reduced secretion showed a clear bias toward low and high (to a lesser magnitude) pI (Fig. 1F). Conversely, proteins that were secreted to higher levels from the Δ*mas* strain showed bias toward moderate pIs (~7 to 9). Analysis of the amino acid distributions of each group revealed that proteins with reduced secretion had more positively and negatively charged amino acids (D, E, K, and R) than the other groups, explaining the bias in pI (Fig. 1H). Interestingly, the opposite trend was observed for polar amino acids. Proteins with increased secretion from the Δ*mas* strain contained a greater proportion of these residues. This might explain why a pI bias was observed, but not a bias in hydropathy, as polar amino acids contribute to GRAVY scores but not pI.

Taken together, these results show that PDIM/PGL-deficient *M. marinum* displays a bimodal distribution of secretion, with an overall unit decrease in secretion. This secretion deficiency is largely nonspecific regarding gene ontology, molecular weight, and hydrophobicity but biased toward proteins with extreme pIs. Charged proteins at physiologic pH specifically require PDIM/PGL lipids for optimal secretion from *M. marinum*. Other systems (including Sec) are likely impacted by the loss of PDIM/PGL, but we are unable to describe them at this time. However, the effects on ESX-1 are specific, significant, and consistent with our model in Cronin et al. (22).

## Data Availability

Raw files are deposited and available at MassIVE (ftp://MSV000093713@massive.ucsd.edu, MSV000093713) and Proteome Exchange (accession numbers PXD048051 and MSV000093713). Search result files used in this analysis can be found in the supplemental information of Jones et al. (23).
